# Correction: Partitioning the Fitness Components of RNA Populations Evolving *In Vitro*

**DOI:** 10.1371/annotation/e97dcbca-dd1b-45cb-a9a0-c2d5c9ff88f3

**Published:** 2014-01-16

**Authors:** Carolina Díaz Arenas, Niles Lehman

Sub-figure 1A is incorrectly omitted from Figure 1. Please view sub-figure 1A here: http://plosone.org/corrections/pone.0084454.g001A.cn.tif

