# Correction: Partitioning the Fitness Components of RNA Populations Evolving *In Vitro*

**DOI:** 10.1371/journal.pone.0096961

**Published:** 2014-05-05

**Authors:** The PLOS ONE Staff

## Notice of Republication

This article was republished on April 3, 2014, to include an image which was missing from Figure 1. Please download this article again to view the correct version. The originally published, uncorrected article and the republished, corrected article are provided here for reference.

## Supporting Information

File S1 Originally published, uncorrected article.
(PDF)

File S2 Republished corrected article.
(PDF)
