# Supplementary material for: Correction: Partitioning the Fitness Components of RNA Populations Evolving In Vitro
Source: PLoS One. 2014 May 5;9(5):e96961. doi: 10.1371/journal.pone.0096961 (PMC4010522; doi:10.1371/journal.pone.0096961)
Supplement: File S1 — Originally published, uncorrected article. (PDF) [file pone.0096961.s001.pdf]

PLOS ONE

Partitioning the Fitness Components of RNA Populations Evolving *In Vitro*

Carolina Díaz Arenas, Niles Lehman*

Department of Chemistry, Portland State University, Portland, Oregon, United States of America

Abstract

All individuals in an evolving population compete for resources, and their performance is measured by a fitness metric. The performance of the individuals is relative to their abilities and to the biotic surroundings – the conditions under which they are competing – and involves many components. Molecules evolving in a test tube can also face complex environments and dynamics, and their fitness measurements should reflect the complexity of various contributing factors as well. Here, the fitnesses of a set of ligase ribozymes evolved by the continuous *in vitro* evolution system were measured. During these evolution cycles there are three different catalytic steps, ligation, reverse transcription, and forward transcription, each with a potential differential influence on the total fitness of each ligase. For six distinct ligase ribozyme genotypes that resulted from continuous evolution experiments, the rates of reaction were measured for each catalytic step by tracking the kinetics of enzymes reacting with their substrates. The reaction products were analyzed for the amount of product formed per time. Each catalytic step of the evolution cycle was found to have a differential incidence in the total fitness of the ligases, and therefore the total fitness of any ligase cannot be inferred from only one catalytic step of the evolution cycle. Generally, the ribozyme-directed ligation step tends to impart the largest effect on overall fitness. Yet it was found that the ligase genotypes have different absolute fitness values, and that they exploit different stages of the overall cycle to gain a net advantage. This is a new example of molecular niche partitioning that may allow for coexistence of more than one species in a population. The dissection of molecular events into multiple components of fitness provides new insights into molecular evolutionary studies in the laboratory, and has the potential to explain heretofore counterintuitive findings.

Citation: Díaz Arenas C, Lehman N (2013) Partitioning the Fitness Components of RNA Populations Evolving *In Vitro*. PLoS ONE 8(12): e84454. doi:10.1371/journal.pone.0084454

Editor: Luis Menéndez-Arias, Centro de Biología Molecular Severo Ochoa (CSIC-UAM), Spain

Received August 27, 2013; **Accepted** November 15, 2013; **Published** December 31, 2013

Copyright: © 2013 Díaz Arenas, Lehman. This is an open-access article distributed under the terms of the Creative Commons Attribution License, which permits unrestricted use, distribution, and reproduction in any medium, provided the original author and source are credited.

Funding: This work was funded by the National Science Foundation (grant DEB-0315286 to NL) and the National Aeronautics and Space Administration (grant NNX07-AU05G to NL). The funders had no role in study design, data collection and analysis, decision to publish, or preparation of the manuscript.

Competing Interests: The authors have declared that no competing interests exist.

* E-mail: niles@pdx.edu

Introduction

The concept of fitness has long been applied to organismal populations as a means to understand their evolutionary dynamics. In the last few decades this notion has been extended to molecular populations that may have preceded cellular life, with both theoretical [1,2] and empirical [3,4] treatments. However, while it is clear that the fitness of organisms can be partitioned into key components such as survival, reproduction, and sexual benefits, this is less clear for populations of life-like molecules such as catalytic RNA (ribozymes) as they evolved on the primitive Earth or in the laboratory [5]. A thorough dissection of how molecular assemblages can experience the different facets of selection pressure would provide a needed link between prebiotic chemistry and organismal biology.

Separating the total fitness of an individual into its different fitness components allows researchers to better assess which selective agents have a stronger impact on the survival of an individual. Darwin was probably the first to separate fitness into fitness components when he studied sexual dimorphism in birds. The components in which the total fitness of an individual can be broken down are chosen on a particular basis, depending on population and ecological dynamics relevant to the survival of the individuals. For example, the total fitness of a bird can be divided in fitness components such as colorful plumage (for partner attraction), singing and dancing (for courtship), and fertility (for breed size).

According to Falconer [6] the selection pressures occurring during the evolution of a population can change the mean fitness value of a phenotypic trait. The extent of this change can be used as a direct estimate of the effect of a specific trait on the total fitness of an individual [6]. Following this line of thought, by knowing the selection pressures acting on each component of fitness one could calculate the total fitness of the individuals in the population. However, in practice this calculation is complicated by various factors: (1) The mean change in the fitness value of a phenotypic trait depends not only on the action of selection on the trait but also on correlated traits, either by pleiotropic effects, or due to linkage disequilibrium [7]. (2) The magnitude and effect of selection on a particular trait can change from one part of the life-cycle to another. (3) The particular selection pressure on a trait depends on ecological dynamics. For example, the relative impact of a component of fitness, such as colorful plumage, on the total fitness of the individual (*e.g.*, the bird) can vary depending on the trade-off between the number of available females that can be attracted by the colorful plumage and number of predators that can easily spot the bird with the colorful plumage [8]. In this scenario, the selection pressure on the trait will be affected by the number of predator present in the environment of the bird.

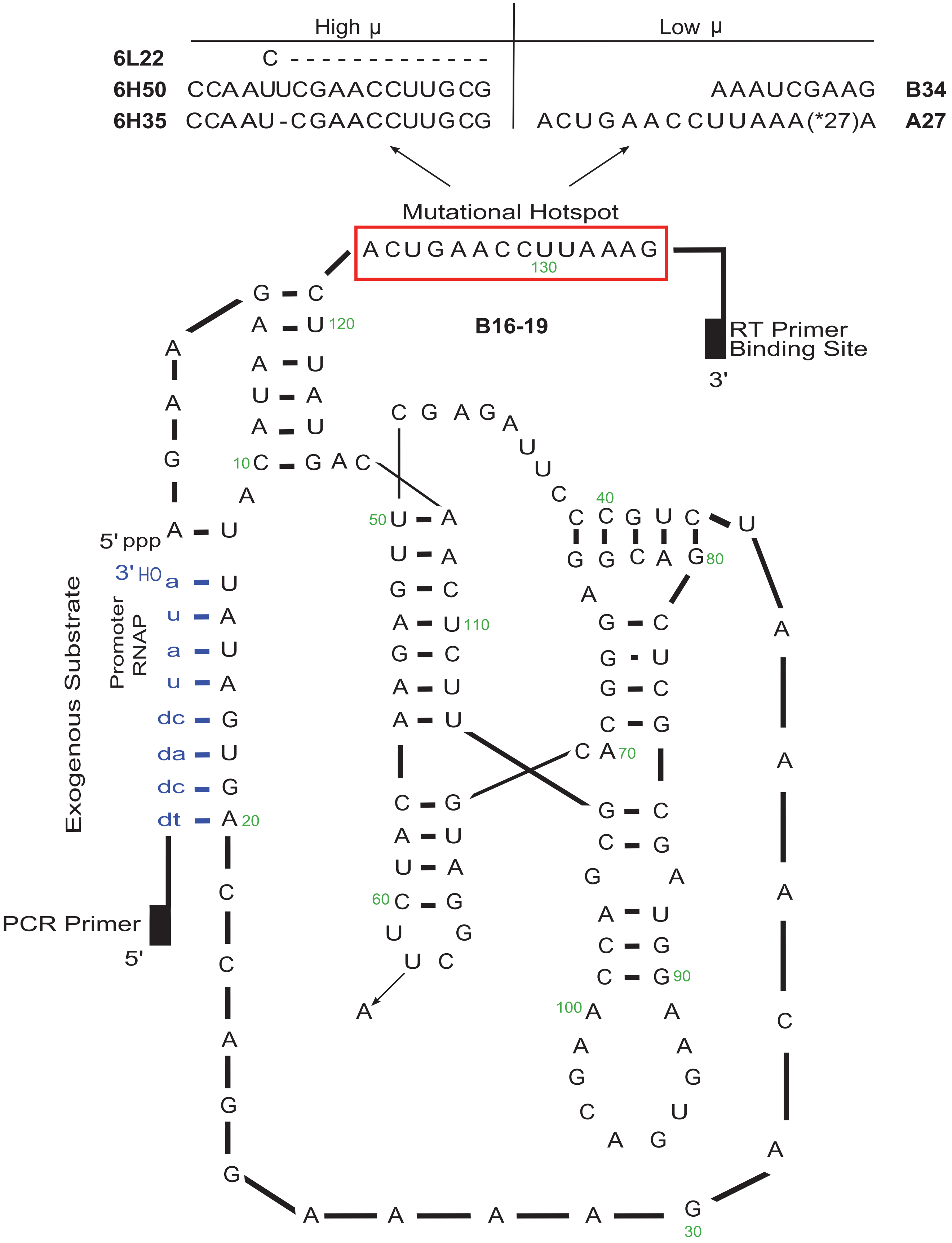

Figure 1. The Continuous *in vitro* Evolution (CE) cycle. (A) Schematic of the CE cycle, which consists of three consecutive enzymatic steps of amplification [12]. Each enzymatic step constitutes a component of fitness. The first enzymatic reaction is the ligation performed by the ligase

ribozyme itself. During this step the ligase catalyzes the ligation of an exogenous substrate to its 5′-end to form a ligase-substrate complex. The second step is the reverse transcription performed by the reverse transcriptase (RT). During this step, cDNA copies are made of both the ligases and ligase-substrate complexes. The third enzymatic step is forward transcription performed by the T7 RNA polymerase (RNAP). Only the ligase-substrate complexes carry the promoter necessary for recognition and transcription by RNAP; cDNA copies of ligases (uncatalyzed) alone are not recognized and represent the end of their lineage (analog to death). (B) Secondary structure representation of the B16–19 genotype, the wild-type ligase used to seed all the evolution cycles, bound to the exogenous substrate (blue, lower case) at its 5′-end. Notice that the promoter for the T7 RNAP is located in the exogenous substrate. Mutations that emerged during the evolution cycles are shown with arrows. The unpaired 3′-end of the ligase has a mutational hotspot where most of the mutations occurred. The mutations that occurred in this region are noted with the name of the mutant ligase genotype that carries them. The U62→A mutation, present in many of the genotypes in this study, is also indicated.
doi:10.1371/journal.pone.0084454.g001

These factors complicate the calculation of fitness effect of each particular phenotypic trait on the total fitness, both in wild and laboratory organismal populations. Approximations have been done using multiple-level and partial regression analysis of fitness variance in order to calculate the effect of selection in separate stages of the life-cycle [9] and to estimate the correlation between selection and relative fitness effects of a particular trait [10,11], respectively. In order to know the absolute fitness of an individual after k episodes of selection, one needs to know the frequency distribution of the phenotypic values and their associated fitness in each round of the selection process. So, if W_k is the absolute fitness of an individual at the kth round of selection, the total fitness (W_T) of the individuals can be equated to the multiplicative effect (Π) of the absolute fitness (W) of all the episodes of selection (k) from 1 to m [9], as follows (1):

$$W_T = \prod_{k=1}^{m} W_k \tag{1}$$

Although measurements of the various fitness components and their multiplicative effect on the total life time fitness of organisms are commonly used, to our knowledge this approach has not being applied to molecular populations. Few molecular evolution experimental techniques allow the molecules to evolve "naturally", with minimal addition of external selection pressures. Yet in continually evolving populations of molecules, that mimic the evolution of organisms in the wild, the total lifetime fitness may indeed be separable into components. In the continuous evolution *in vitro* (CE) system [12], for example, the total fitness of the individual molecules is affected by the rate of all the individual enzymatic reactions participating in the amplification process of the molecules. Each of these steps can be considered a component of fitness and thus the CE system has great potential to explore the effect of different selection pressures on each component of fitness and the consequences on the total fitness of the individuals. As the selection dynamics on molecular populations are similar to those of natural populations [5,13], the experimental evidence gathered on molecular populations will facilitate the understanding of the same dynamics on natural populations.

The CE system [12] uses catalytic RNA (ligase ribozymes) molecules as the subject of study because the effects of mutations acquired during their laboratory evolution cycles can be directly measured in their phenotype [14,15,16]. As cycles of evolution occur, mutations arise and accumulate, having differential effects on the phenotypic traits of the ribozymes. Some mutant ribozymes eventually become frequent enough to allow their detection by direct sequencing, rendering *a posteriori* measurement of the change in fitness value of the evolved individuals possible. Mutations arise in the CE system as a result of enzymatic mistakes made during the amplification process of the molecules. The CE system (Figure 1A) is composed of three different enzymatic steps:

1) The first is the ligation step performed by an RNA enzyme. The ligase ribozyme catalyzes the reaction that occurs by attack of the 3′-OH of a trans substrate onto the 5′-phosphate of the ligase itself with consequential formation of the respective phosphodiester linkage. The ligase-substrate complex formed carries a 17 nucleotide (nt) promoter sequence for the T7 RNA polymerase.
2) The second is the reverse transcription, performed by MMLV reverse transcriptase (RT), an RNA-dependent DNA polymerase. This protein enzyme uses the RNA ligase as a substrate to synthesize complementary DNA copies. Copies of ligases that have reacted (ligase-substrate complex) and that have not reacted (ligase per se) are both made by the RT. This enzyme is error-prone because it lacks a 3′→5′ proof-reading ability [17]. Therefore as cycles of evolution occur, mutations accumulate, unless there is some mechanism to alleviate them (i.e., selection).
3) The third is (forward) transcription, performed by T7 RNA polymerase (RNAP), a DNA-dependent RNA polymerase. This enzyme synthesizes RNA copies that are complementary to the cDNA strand, when it recognizes the promoter sequence. Because the promoter sequence is located in the 3′-end of the exogenous substrate (Figure 1B), only reacted ribozymes posses this promoter necessary for the RNAP to initiate transcription. Unreacted ligases are not recognized by this enzyme and they represent the end of their genotype's evolution (analogous to death).

Here, we evaluated the fitness components of ligase genotypes that resulted from several parallel lines (lineages) of CE initiated from a pure population of a single RNA species, the B16–19 genotype (Figure 1B) [18], which is a high-fitness 152-nt ribozyme that has emerged from several previous CE studies [3,18,19,20]. Lineages seeded with this ligase can evolve through spontaneous mutations imposed by the protein enzymes RT and RNAP, and new genotypes of higher, neutral, or lower fitness can be rapidly driven to fixation when small population sizes are used ($N = 3000$ or less; see Table 1). To quantify the total fitness of the ligases evolved during the CE cycles, we estimated the rates of reaction of each of the three aforementioned enzymatic steps (Figure 1A). For the RNA ligation step we used the Eadie-Hofstee linearization method [21] to measure k_{cat}, and, for the RT and RNAP enzymes, we measured product formation over time. Each step constitutes a component of the total lifetime fitness of the ligase evolution. We now present the first systematic study of fitness components at the molecular level.

Materials and Methods

Measuring Fitness Through RNA Reaction Rates

The rates of enzymatic reactions can be measured in the laboratory in different ways. One practical way is to measure the product formation over time. A plot of product formation over time is drawn, and the rate can be inferred from the slope of the

Table 1. Lineages evolved with the CE system.

**Mutation rate**	N_e					
	**100 molecules (167 yochtomoles/8.2 μL)**			**3000 molecules (5 zeptomoles/8.2 μL)**		
	**Name**	**t_E (obs)**	**E[t_E]**	**Name**	**t_E (obs)**	**E[t_E]**
MMLV-RT:	3Z	18	24.3	2I	36	44.5
1/30,000	4R	18		2K	37	
	4U	34		2A	45	
	**4V**	18		2J	49	
	4W	30		3A	50+	
	4Y	28		**3C**	50+	
MMLV-RT+Mn^{2+}:	6E	50	50	6B	50	50
6–30 fold higher than 1/30,000	**6H**	50		6N	50	
	6K	50		**6O**	50	
	**6L**	50		6P	50	

The effective size (N_e) of the populations evolved is given in the top row of the table, and the approximate mutational rate (μ) used is given in the first column. These mutational rates are based on literature review [30–33]. In the body of the table is the data for the two population sizes used (100- and 300-molecule), as follows: name of the lineages in the first column, observed time to extinction (t_E) in the second column, and mean time to extinction time in the third column, as an estimate of the expected time to extinction (E[t_E]). All the replicate lineages were evolved under the same conditions, and the evolution history can be reviewed in [24,26]. The replicate lineages that were studied in depth for population diversity and genotypic characterization, and from which the mutant ligases were selected (based on relative high frequency) to study the fitness are shown in bold. The emergence of these mutants in the populations had a direct evolutionary implication in the fate of their lineages.
doi:10.1371/journal.pone.0084454.t001

curve in simple cases. If the rate of the reaction is not linear, there are several methods that can be used to convert the data into its linear representation to gain a more clear representation of the true enzymatic rate. Also, many physical variables play important roles in the rates of reaction of enzymes. For this reason we measured the reaction rates under the same conditions that are used during the CE experiments to the best of our abilities. In the case of fast enzymatic reactions (*e.g.*, RNA-catalyzed RNA ligation) some variables such as temperature or pH can be varied in order to decelerate the reaction [22].

We measured all the reactions using the CE buffer as described previously [23,24]. At its final working concentration, the buffer contains 50 mM KCl, 25 mM $MgCl_2$ and 30 mM EPPS (pH 8.3). All enzymatic reactions were stopped using a quench solution consisting of 2× polyacrylamide gel-loading dye (0.05% bromophenol blue and 40% sucrose) and 25 mM Na_2EDTA.

The ligases were prepared differently depending on which enzymatic rate was to be measured, but the protein enzymes were employed at the same concentration as during all CE experiments: 9.6 U/μL for MMLV-RT (USB Corp., Cleveland OH) and 2.4 U/μL for T7 RNAP (Affymetrix/Ambion, Austin TX). The following sections detail the methods for measuring the kinetics of each enzymatic step.

Ligase Kinetics: Preparation of the Ligase Ribozymes

DNA products of all ligase ribozymes evolved during the CE were transcribed *in vitro* using Ambion T7 RNA polymerase at a final concentration of 2 U/μL, 2 mM each rNTP, and transcription buffer (15 mM $MgCl_2$, 2 mM spermidine, 5 mM dithiothreitol (DTT), and 50 mM Tris pH 7.5). The transcription products were purified by electrophoresis through 8% polyacrylamide/8 M urea gels, and the bands were excised following a "Dip-N-Dot" procedure [25]. Ligase transcripts were adjusted to 10 μM using UV spectrometry at 260 nm by careful dilution. From this "ligase stock", a mixture (10 μL) was prepared for each ligase concentration destined to be used for the kinetics assays. The amount of 3.33× CE buffer was held constant (3 μL) while the amount of stock (1.0 μM) ligase and of RNase-free water (Ambion) varied as the desired final concentration of ligase varied. Nine concentrations for each of the six ligases were prepared: 0.050, 0.075, 0.100, 0.150, 0.200, 0.250, 0.500, 0.750, and 1.000 μM.

Ligase Kinetics: Preparation of the Substrate for Continuous Evolution

The substrate for the ligase ribozyme, S-163, is a DNA/RNA chimera that has the 17-nt T7 RNA polymerase promoter sequence (underlined): 5′-CTTGACGTCAGCCTGGACTAA-TACGACTCACrUrArUrA-3′. For the ligase kinetics, 1 μM substrate was radiolabeled at the 5′-end using 1.26 μM γ-^{32}P-ATP (2,280 Ci/mmol) with 1× OptiKinase reaction buffer from USB Corp. (50 mM Tris-HCl pH 7.5, 10 mM $MgCl_2$, 5 mM DTT), and 0.5 U/μL OptiKinase^{TM} enzyme (USB Corp.) The reaction was incubated for 1 h at 37°C. Radiolabeled substrate was purified on a 15% polyacrylamide/8 M urea gel for 2000 V*h and the bands were excised following the Dip-N-Dot procedure [25]. The product was calibrated for concentration with a UV spectrophotometer at 260 nm. This stock substrate was used to prepare the substrate mix for the ligase kinetics, containing 10 nM radiolabeled S-163 and 1× CE buffer.

Ligase Kinetics: Ligation Reactions

The rate of the ligation reaction between each ligase concentration and the substrate was measured by performing a series of six time-point reaction samples from 5 sec to 2 min in a V-bottomed 96-well plate. The substrate mix was placed in row number 1 of the plate (the "reaction-well") in all columns, each one (from A to L) used for a different ribozyme concentration. One plate per enzyme was used. Quench solution was added to the "quench-wells" from row numbers 3 to 8. A laboratory timer was set for 2 min, 10 sec and initiated. When the timer reached 2 min, the ligase mixture was added to the "reaction-well" in a 1:1 volume ratio with the substrate mixture. This ensures a $MgCl_2$ concentration of 25 mM and at least 10-fold enzyme concentration relative to the substrate. For each time point, an aliquot of the

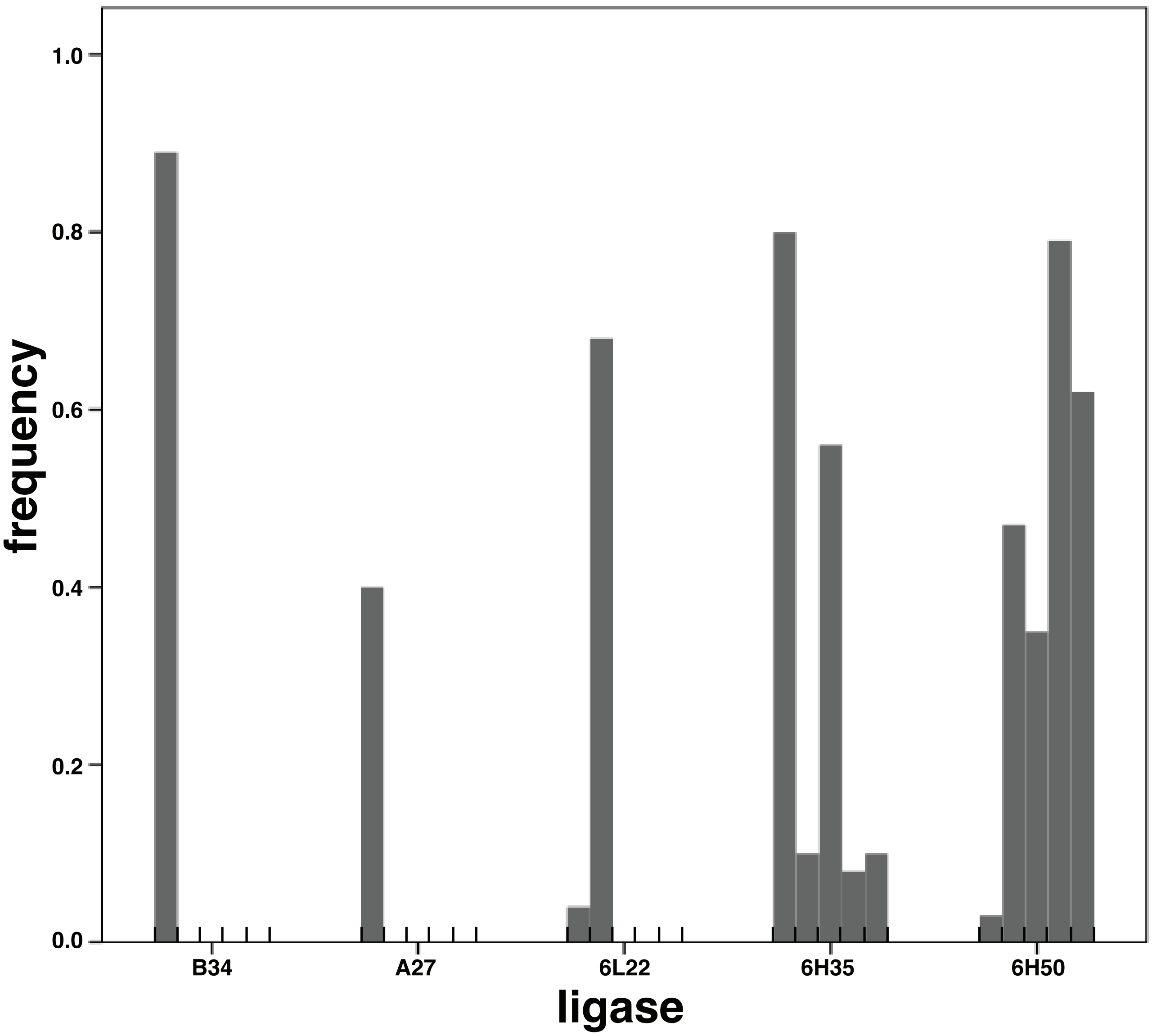

Figure 2. Bar diagram showing the frequency at which ligase ribozyme mutants appeared in the different lineages that they were observed. Each bar represents a burst that was evaluated [24,26]. Overall it can be seen that the frequencies of these mutants in the different bursts studied is over 40%. The name of the mutants is given according to the lineage and/or burst in which they emerged, with the exception of A27. Note that B34 emerged from a 3000-molecule bottleneck-size lineages [26] as opposed to the other mutants that are from a 100-molecule bottleneck-sized lineage.
doi:10.1371/journal.pone.0084454.g002

ligase-substrate mixture was removed from the reaction-well and was added to the quench-well in a ratio of 1:1, to get an EDTA concentration of 25 mM.

The reaction products were then electrophoresed on 8% polyacrylamide/8 M urea gels for 500 V*h to separate the reacted ligases (187-nt fragments) from the residual substrate (35 nt) fragments. Each time point was loaded in a single well of the gel, and all the time points for the same enzyme concentration were loaded in the same gel to account for potential gel irregularities that may affect the readings. The gels were exposed to a phosphor screen overnight and visualized on a Typhoon imager (GE Healthcare). The fraction of product was quantified using ImageQuant software (GE Healthcare). Plots of the fraction reacted *vs.* time were drawn using KaleidaGraph v. 4.1 (Synergy Software). The data were fitted following the equation $y = A(1- e^{-kt})$, where A is the asymptote and k is the initial slope of the curve. The k_{obs} obtained for each enzyme concentration were plotted following a modified Eadie-Hofstee method [19,21]. The k_{obs}/[ligase] vs. k_{obs} plots were drawn using SPSS v.21, and the k_{cat} and K_m values were obtained from the y-intercept and the slope of line, respectively.

Table 2. Absolute rates of the enzymatic reactions for each ligase, for each fitness component (ligation, reverse transcription and transcription).

Ligase	Ligation	Reverse transcription	Transcription	Total Fitness	Rank
B16	10.76	5.737×10^{4}	1167	7.206×10^{8}	3
A27	0.85	5.724×10^{4}	1293	6.302×10^{7}	6
B34	18.22	7.006×10^{4}	1378	1.759×10^{9}	1
6L22	1.59	4.212×10^{4}	1272	8.519×10^{7}	5
6H35	13.32	6.144×10^{4}	729.3	5.969×10^{8}	4
6H50	10.08	1.316×10^{5}	847.7	1.124×10^{9}	2
C.V.	0.74	0.45	0.24		

Ligation rates are in turnovers *per* minute, transcription and reverse transcription rates are in amount of product (cDNA and RNA, as phosphorimager band concentration units) formed *per* minute. The total fitness for each ligase is obtained by multiplicative effects of each fitness components. The coefficient of variation (C.V.) is given in the last row for each fitness component.

doi:10.1371/journal.pone.0084454.t002

MMLV-RT Kinetics: RNA Preparation

Ligases were transcribed *in vitro* as described above under the ligase kinetics section. The transcripts were diluted to approximately the same concentrations. The reverse transcription primer was radiolabeled following the same procedure that was used to radiolabel the substrate S-163. The radiolabeled primer was purified in a 20% polyacrylamide/8 M urea gel for 2400 V*h, and the band was excised following the Dip-N-Dot procedure [25]. The concentration of the homogenized primer product was measured with UV photometry at 260 nm. The concentration of the radiolabeled primer was calibrated by careful serial dilution to 0.3 μM.

Table 3. Relative values of the rates of enzymatic reactions in relation to the ligase with highest value of fitness (1.00).

Ligase	Ligation	Reverse transcription	Transcription	Total
B16	0.59	0.44	0.85	0.41
A27	0.05	0.44	0.94	0.04
B34	1.00	0.53	1.00	1.00
6L22	0.09	0.32	0.92	0.05
6H35	0.73	0.47	0.53	0.34
6H50	0.55	1.00	0.62	0.64

The relative fitness for each ligase is obtained by the rate of each ligase over the highest value for each component. The ligases with the highest values are B34 (ligation, transcription, and total fitness), and 6H50 (reverse transcription). See Figure 4 for visual comparisons.

doi:10.1371/journal.pone.0084454.t003

MMLV-RT Kinetics: Reverse Transcription Rate Measurement

The clean RNA ligases were mixed with a dNTP mix (dATP, dTTP, dCTP, and dGTP, each at 25 mM at 1×), 0.6 μM radiolabeled primer (3521 Ci/mmol), and 1× CE buffer. The reaction rate was measured by taking time points of the reaction from 3 to 21 min. The timer was set to 25 min and initiated. At minute 24, 9.6 U/μL MMLV-RT enzyme (USB Corp.) was added to the reaction vessel. At minute 21, the first aliquot was taken from the reaction vessel and quenched. In the same manner, every three minutes an aliquot is drawn from the reaction vessel and quenched into the respective time point quench solution. In total seven time points were taken.

The reverse transcription products from each time point were loaded in a different well, and electrophoresed into 8%

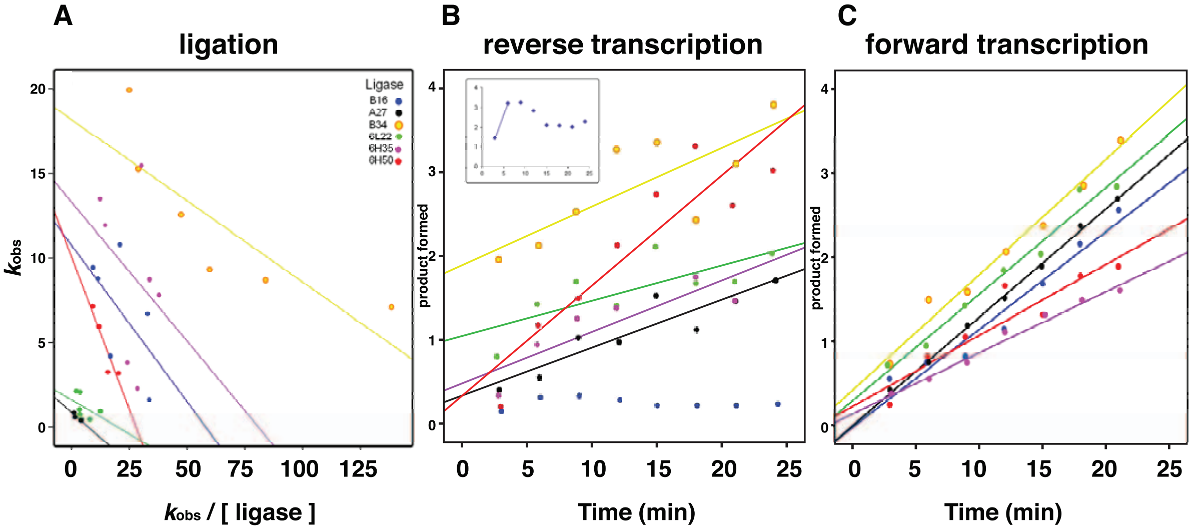

Figure 3. Plots for the three enzymatic rates of reaction, ligation, reverse transcription and transcription. (A) ribozyme-catalyzed ligation reaction. The ligation reaction was measured for each ligase ribozyme as amount of fraction reacted per time, and was linearized following an Eaddie-Hofstee method; ligase color-coded data points are shown with their corresponding linear fit line. The k_{cat} estimate of the reaction rate is obtained from the slope of the fitted line in the k_{obs}/[Ligase] vs. k_{obs} plots. (B) Reverse transcription reaction. This enzymatic step was measured as the amount of product formed (in concentration of phosphorimager band units) over time (minutes) for each ligase. Ligase color-coded data points are shown with their corresponding linear fit line. The estimate of the rate is obtained from the slope of the fitted line. (C) Transcription reaction. This enzymatic step was measured as the amount of product formed over time (in concentration of phosphorimager band units) over time (minutes) for each ligase.

doi:10.1371/journal.pone.0084454.g003

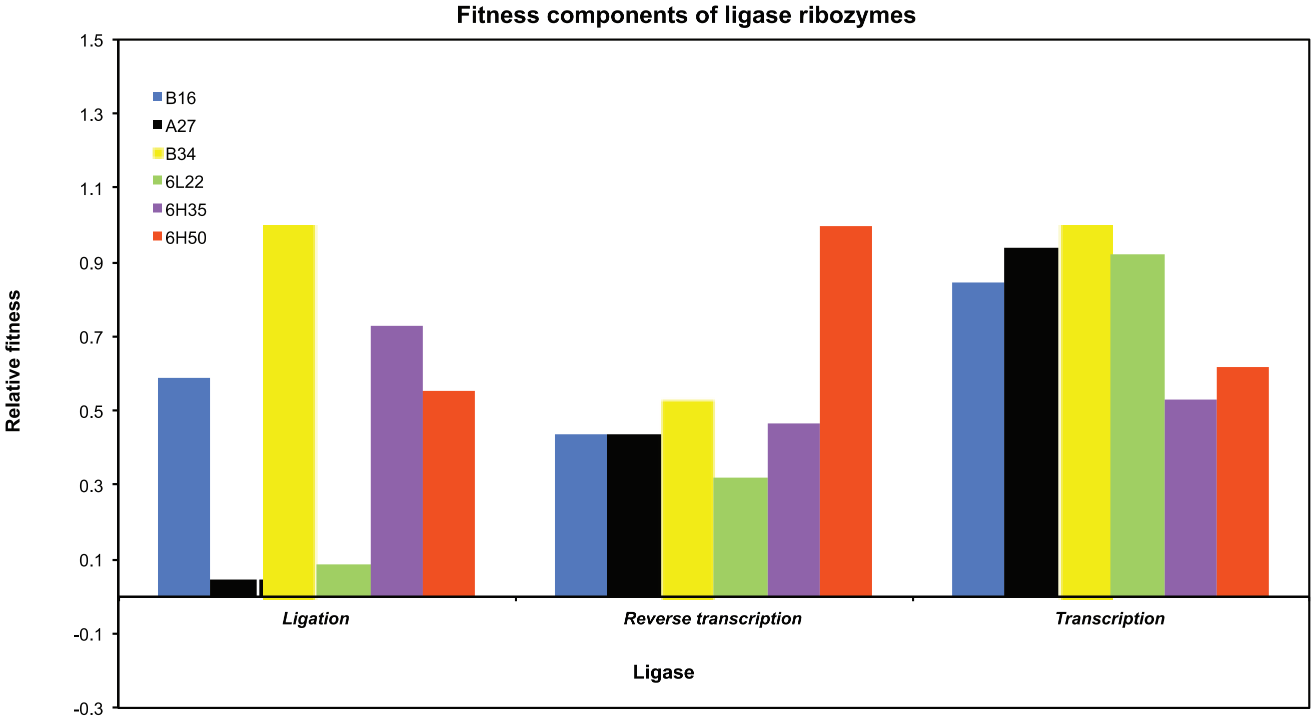

Figure 4. Component of fitness of the ligase ribozymes. The three enzymatic steps of ligation, reverse transcription, and transcription were measured for each ligase. The strength of the fitness components (*x*-axis) over the relative fitness (*y*-axis) is depicted here for each ligase by the height of the ligase-color-coded columns. Notice that different genotypes have particular strengths in different fitness components. For ligation, A27, 6L22 and B34 have extreme values; for reverse transcription, 6H50 has an extreme value; and for transcription, 6H35 has a slight low value.
doi:10.1371/journal.pone.0084454.g004

polyacrylamide/8 M urea gels, which were exposed overnight to phosphor screens. One gel was prepared for the reverse transcription of each ligase. The gels were visualized and quantified as described above. Plots of the product formed *vs.* time were drawn using SPSS v.21. The units are in "concentration of phosphorimager band" *per* minute, but are given as *per* minute in the Figures and Tables and in the Results and Discussion section, for simplification.

RNA Polymerase Kinetics

The rate of the forward transcription reaction was measured for each ligase ribozyme, by mixing clean PCR product with 1× rNTP mix (UTP, CTP, GTP, and ATP, each at 2 mM), 1× CE buffer, 0.1 μM ^{32}P-γ-ATP (6000 Ci/mmol). The rates were measured by taking time points as done for the reverse transcription rate measurements. Time points were taken every three minutes for a period of 21 minutes, which is equivalent to the length of one "burst" of RNA production during the CE experiments. (One burst represents about three generations in Figure 1A.) Once the timer reached minute 24, 2.4 U/μL of enzyme T7 RNA polymerase (Ambion) were added to the reaction vessel. Time points were taken by removing an aliquot from the reaction mix and quenching it in a 1:1 quench solution as described above.

The reaction products were loaded onto 8% polyacrylamide/8 M urea gels, electrophoresed for about 1400 V*h, and subjected to phosphorimaging as described above. Plots of the product formation *vs.* time were created in SPSS v.21, and the rate was obtained from the slope of the line. The units are in "concentration of phosphorimager band" *per* minute, but are given as *per* minute in Table 1 and in the Results and Discussion section, for simplification.

Calculation of Multiple Fitness Components

The rates of the ligation, the forward transcription, and the reverse transcription reactions were used as fitness component of the ligases. To calculate the total fitness value for each ligase, a multiplicative procedure following Arnold and Wade [9] was used. The relative fitness value was calculated as a rate of each ligase fitness over the ligase with the highest fitness value, for each component of fitness. The correlation plots were made in Microsoft Excel v.14.

Results and Discussion

Ligases Evolved during CE

During the evolution of lineages initiated with the ligase B16–19, new genotypes emerged *via* spontaneous mutation and persisted long enough in the population to be detected by direct nucleotide sequence analysis [24,26]. During the evolution experiments in which a low mutation rate was used, two ligase genotypes emerged with high enough frequency to be chosen for our fitness assays. These two ligases have been previously evaluated for their k_{cat} values [26]. During subsequent evolution experiments in which the mutation rate was increased by adding Mn^{2+}, three new ligases emerged [24]. Thus, in the experiments described here, these six ligases (Figure 1B), including B16–19 (the "wild-type"), were evaluated for their fitness profiles. The name of each ligase is derived from the line and burst in which they were arose during their original continuous evolution cycle (Figure 1A). The following is a short description of each ligase with its respective name:

1) **B16–19**: the ligase ribozyme used to seed the CE experiments. It was selected to initiate the lineages because it has

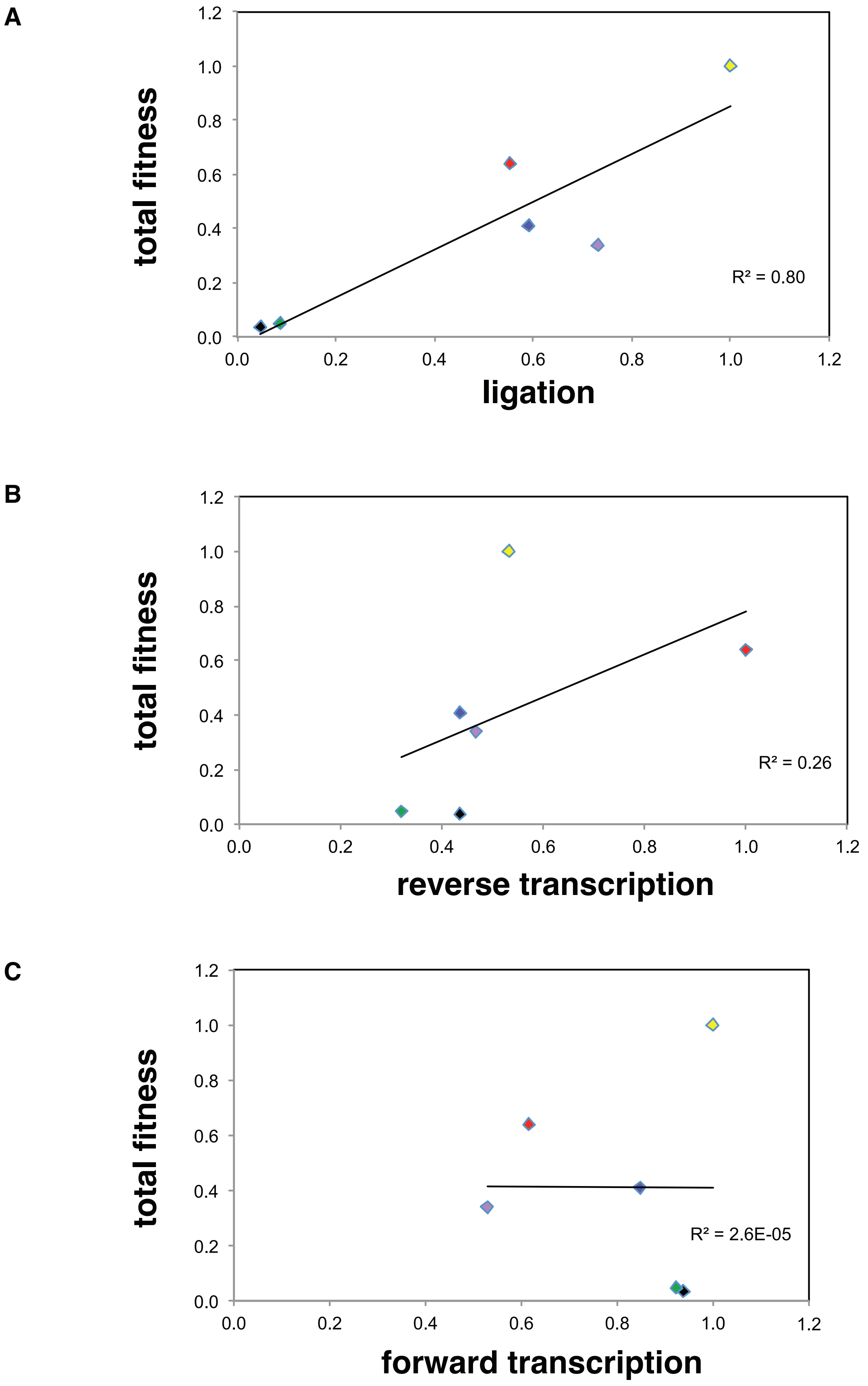

Figure 5. Correlations between the total fitness and each of the fitness components as an estimate of the impact of each parameter on the total fitness. Linear fits to the data are shown for (A) ligation, (B) reverse transcription and (C) transcription, with their corresponding R^2

values. The ligation parameter is correlated to the total fitness with an R^2 value of 0.80, while the other two parameters (reverse transcription and forward transcription) are not, indicating a disproportionate influence of the ligation on the total fitness.
doi:10.1371/journal.pone.0084454.g005

one of the highest catalytic rates known for a ligase ribozyme, as previously measured (13 min^{-1}) [26] and hence a relatively high fitness value. Mutations that arise in the system are more likely to have a deleterious effect on the reaction rate of the ligase and its fitness value would decrease [20]. As mutations accumulate, the mean fitness of the population can decrease, potentially driving the population to extinction. The sequence at the 3′-end of this ligase: **5′–(120)- UCACUGAAC-CUUAAAG–(135)-3′** was found to contain the main variations among the following mutant ligases.

2) **A27**: a ligase evolved during the experiments that employed no added mutagen [26]. This ligase is characterized by a long poly-A tail and has been observed in lineages that became extinct as a consequence of the onset of Muller's Ratchet and further mutational meltdown [26]. This ligase actually represents a group of mutants with poly-A tails on the 3′-end of the ligase that can have different lengths, from a few nucleotides to several (or even over a hundred). For example, an A5 mutant has five additional A's, as follows: **5′–(120)-UCACUGAACCUUAAAAAAAAG-(140)–3′**. The rate constant of a mutant (A23) from this group was previously measured (6 min^{-1}) [26]; and found to have a deleterious effect on the population. The poly-A ligase characterized in this study has 27 additionally A's.

3) **B34**: a ligase that also emerged during the no added mutagen studies [26]. This ligase, in contrast to those containing a poly-A tract, was never observed in lineages that went extinct. The ligase B34 was observed in lineages that evolved for 50 cycles or nearly so. The rate constant k_{cat} of this ligase has been previously measured (18 min^{-1}) [26]; and thus it was found to be an advantageous mutation. This ligase is characterized by the following sequence variation at the 3′-end: **5′–(120)-UCAAAUCGAAG-(130)–3′**, plus a nucleotide substitution at the position 62 (U62→A).

4) **6L22**: a ligase evolved during the high mutation rate experiments [24]. It is the least frequent of all the six ligases evolved during our CE experiments and is usually observed at the beginning of the lineages, being displaced out of the population by other ligases. It is characterized by a short sequence deletion at the 3′-end of the ligase: **5′–(120)-UCC-**

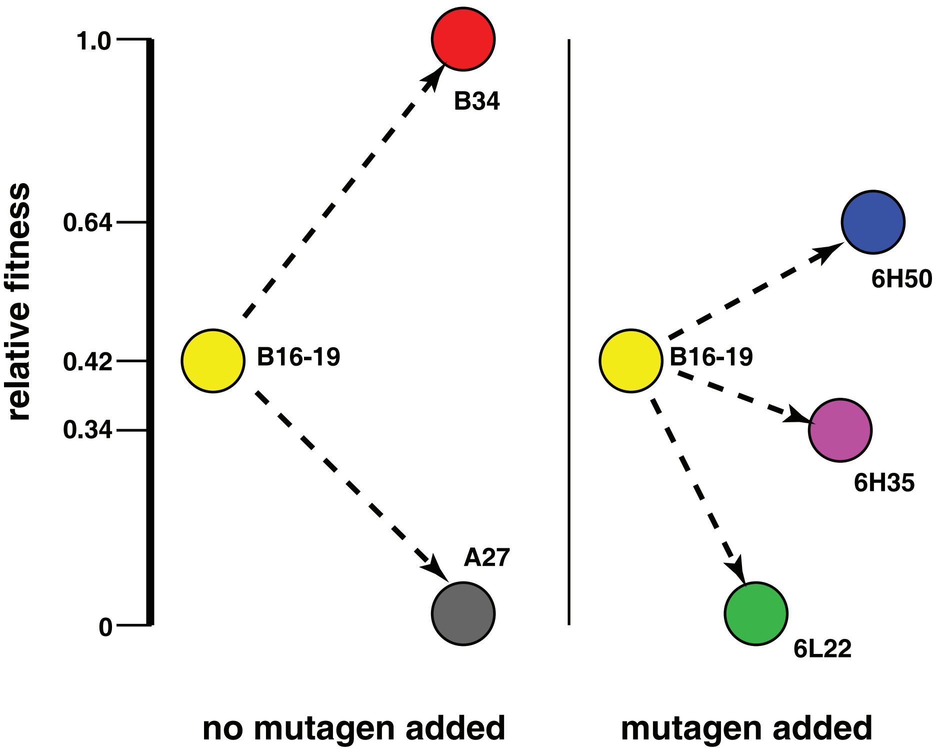

Figure 6. Fitness of the ligases and their relationships in crude evolutionary time. The evolution experiments [24,26] start with a homogeneous population of the B16–19 ribozyme (yellow). As evolution cycles go (*x*-axis), mutant ligases emerge with different total fitness values (*y*-axis). Polymorphic populations can persist through time or go extinct depending on the mean fitness value and the mutation rate. Ligases A27 and B34 evolved from B16–19 in experiments with no $MnCl_2$ added to the reaction vessel [26] (left), whereas the evolution of 6L22, 6H35 and 6H50 occurred with $MnCl_2$ added to the reaction vessel to enhance the mutation rate [24] (right). The exact evolutionary path followed by an emergent mutant from the wild-type is not known, but the genetic relationship of these mutants in time is known, and depicted here as dotted arrows. Populations where poly-A mutants (*e.g.*, A27) are present in high frequency decrease in size, and are at risk of extinction, due to the low mean fitness value of the population, which keeps decreasing as adenylation tracks are extended. The mutant 6L22 has a relative low value of total fitness, but it emerged in populations evolved at high mutation rate, in which a quasispecies were observed. This is perhaps evidence of the "survival-of-the-flattest", a phenomenon that has been shown to be associated with quasispecies [27–29]. In this case, 6L22 was quickly replaced by 6H35 and 6H50 with higher fitness values. Quasispecies allowed beneficial mutations to emerge, even at the low population size used.
doi:10.1371/journal.pone.0084454.g006

(122)–3′ (Figure 1B). This ligase also has the U62→A mutation.

5) **6H35**: a ligase evolved during the high mutation rate studies as well [24]. The sequence at the 3′-end of the ligase is **5′–(120)-UCCCAAUCGAACCUUGCG-(137)–3′**. It has the mutation U62→A. This ligase has a relatively high frequency compared to other ligases evolved during the mutagenic experiments.
6) **6H50**: a highly dominant ligase in the populations evolved with high mutation rate [24]. It usually emerges towards the end of the evolution path of the populations and rapidly increases in frequency. This ligase has a sequence similarity to 6H35, with only one nucleotide difference: **5′–(120)-UCC-CAAUUCGAACCUUGCG (138)–3′**. It has the U62→A mutation as well.

These aforementioned ligases represent the most frequent ligases that have been observed in prior evolution experiments [24,26], as a result of directional selection (*e.g.*, B34), strong random drift (*e.g.*, poly-A mutants), or quasispecies (6L22, 6H35, 6H50). Although many other ligases have evolved during the CE experiments (Table 1), they were not selected for the current kinetic studies because none of them achieved a relatively high frequency in the previous experiments, although as a group they represent a good fraction of the population mean fitness. The ligases selected for study here must have attained a high frequency (Figure 2) as a consequence of their net fitness values; therefore, the catalytic rates of these ligases serve as good references for the incidence of each catalytic step on the total fitness of the ligases.

The Rates of the Enzymatic Reactions of the CE

The three enzymatic-step components of ligase fitness were measured under environmental conditions very similar to the CE system. The ligation reaction was performed at a lower temperature to facilitate an accurate measurement of the rate by manual pipetting. Previous studies have shown that B16–19 catalyzes 13 turnovers *per* minute [26], and pipetting 10 or more times per minute is not only very challenging but may also render inaccurate estimates of the rate of reaction [22]. The lower temperature results in an underestimated value of the ligation rate compared to the rate during the CE experiments. Still, this measurement allows for a comparison among the rate constants of the various ligases evolved in the system.

Catalytic Rate of the RNA-catalyzed RNA Ligation

Given the fast nature of the ligase reaction, we chose concentrations carefully to measure the kinetic constants. Based on preliminary data (not shown), nine concentrations of ligases were chosen below the upper bound limit of 1.0 μM. Seven time points of product formation were taken from the reactions and the k_{cat} values obtained from the slope of the curve of a modified Eaddie-Hofstee plot traced for each ligase (Figure 3A). The resulting kinetic parameters are also summarized in Table 2.

The B34 ligase has the highest k_{cat} value, with 18 min^{-1}. This value coincides exactly with the one published previously [26], and is not surprising because this ligase has been detected in lineages that survived to burst 50 or nearly so. This mutant evolved in population sizes of 600 and 3000 molecules, which are large enough to allow for natural selection to operate in our CE system. This high catalytic rate may give B34 a replicative advantage in the first step of the reaction cycles. The B16–19 ligase has a k_{cat} value of 10.76 min^{-1}, close to the previously measured value of 13 min^{-1} [26]. These two values are comparable, and given that in this study we used more time points to calculate the intercept, we choose to use this value in subsequent analyses. Although the rate of the reaction of B16–19 is not as fast as B34, this is still a rapid reaction rate and it explains why B16–19 is a highly recurrent ligase during many CE experiments [5,20].

The A27 ligase was found to have the lowest rate constant value, with 0.85 turnovers per minute. This value is lower (6.6 min^{-1}) than the previously measured mutant with 23 extra A's [26]. More A's added to the 3′-end of the ligase decrease its catalytic activity and thereby have a deleterious effect on the population, as previously proposed [26]. Additionally, the extra A's added to these mutant ligases may have a deleterious effect on the population as they can cause a depletion of ATP nucleotides from the pool which could otherwise be used by other ligases.

Ligases 6H35 and 6H50 have rate constant values of 13.32 min^{-1} and 10.08 min^{-1}, respectively. These values are very close to that of B16–19, indicating that, at the first stage of the evolution cycle, ligases 6H35 and 6H50 are as competitive as the wild-type B16–19. Ligase 6L22 has a rate constant of 1.59 min^{-1}. This low value may be the cause of its relative low frequency compared to 6H35 and 6H50, the other ligases evolved during the high mutation rate experiments that developed a quasispecies structure [24]. Ligase 6L22 emerged early in the lineages and was quickly replaced by 6H35 and 6H50, which have higher ligation rates.

Catalytic Rate of the Reverse Transcriptase

We next measured the catalytic activity of the reverse transcription step for each ligase (Figure 3B, Table 2) and found that it also has differential effects on the fitness of each ligase. Ligase 6H50 has the highest value, with 1.316×10^{5} min^{-1}. It is reverse transcribed almost twice as fast as the second fast ligase, providing a head start of the next catalytic step, and an advantage in the use of the dNTP pool. Ligase B34 follows with 7.006×10^{4} min^{-1}, a high value as well. The other ligases have lower values given in decreasing order as follows: 6H35, B16–19, A27 and 6L22 with 6.144×10^{4} min^{-1}, 5.737×10^{4} min^{-1}, 5.724×10^{4} min^{-1}, and 4.212×10^{4} min^{-1}, respectively. Ligases A27 and 6L22 have relatively similar values to B16–19 but the difference in the ligation reaction (compare 0.85 min^{-1} and 1.59 to 10.76 min^{-1}, respectively) is large. Thus, the negative correlation between the first two components of fitness of A27 and 6L22 will likely result in ligases that are reverse transcribed before they ligate the substrate to themselves, and hence will not be recognizable by the T7 RNAP and will not be transcribed.

Catalytic Rate of the Transcription

Next, we measured the forward transcription reaction rate for each ligase as a substrate of the T7 RNA polymerase and again we found notable differences across the genotypes (Figure 3C, Table 2). B34 has the highest rate of forward transcription, with 1378 min^{-1}. This ligase is transcribed more quickly than any other ligase present in the population, which confers it with an advantage over the other ligases. Ligase A27 follows with an also high transcription rate value, 1293 min^{-1}. Ligases 6L22, B16–19, 6H50 and 6H35 have values of 1272 min^{-1}, 1167 min^{-1}, 847.7 min^{-1}, and 729.3 min^{-1}, respectively. Ligase 6L22 has a higher rate of transcription than 6H35 and 6H50, this would give the 6L22 cDNA ligase-substrate complexes an advantage in the competition with 6H35 and 6H50 for resources such as NTPs, however, the rates of ligation and reverse transcription are higher for 6H35 and 6H50 than for 6L22, providing them with an overall advantage, as observed in the quasispecies [24]. The high rate of transcription of A27 and 6L22 could be the cause of their low but

still observable frequency in the populations where they were present.

Total Fitness

Using the absolute values of each catalytic step as a component of fitness, we calculated the total fitness (Table 2) as the multiplicative effect of all the fitness components [9] and ranked them in decreasing fitness values to establish their fitness relationships. Relative fitness values were calculated as the rate between the absolute fitness of each ligase and the highest fitness value for each component of fitness (Table 3). The relative values facilitate the comparison and easy visualization of the different scales among the reaction rates (Figure 4). The relative fitness can be summarized follows: B34 has the highest value (1.0) of fitness and the only one above the 75% percentile. B34 had a beneficial effect on the populations, preventing them from becoming extinct. 6H50, 6H35 and 6L22 have relative fitness values of 0.64, 0.34, and 0.05, respectively, which correspond to their frequencies in the quasispecies [24]. 6L22 is a deleterious mutant, which is quickly replaced by 6H35 (with higher fitness) in the high mutation rate populations. B16–19, the wild-type ligase used to seed all the experiments, has a relative fitness of 0.41. A27 has a relative fitness of 0.04. This deleterious mutant, observed in low-mutational-rate populations, caused extinction due to Muller's ratchet and mutational meltdown [26].

The total fitness values (Table 2) cannot be taken as a direct measure of their performance at the different stages of the evolution cycle. For example, ligase B34 has the highest total fitness but is not the best ligase during the reverse transcription rate; its value is lower than 6H50 for this step. Likewise, each components of fitness has profound implications in survivorship or replicative ability of each ligase (Figure 4), and are free to vary within the total fitness value. The extent of this variation should be indicative of which component(s) is (are) driving evolution. For example, the coefficients of variation for each fitness component, 0.74, 0.45 and 0.20 (ligation, reverse transcription and transcription, respectively), show the ligation components as a more distinctly selectable factor in the total fitness, than the reverse transcription and transcription components, probably giving the RNAs the bulk of their evolutionary edge. For ligation, A27, L22 and B34 have extreme values (Figure 4); for reverse transcription, H50 has an extreme value; and for transcription, H35 has a slightly lower value. Similarly, the correlation of the total fitness with each of the fitness component (Figure 5) shows that the ligation ($R^2 = 0.80$) play a major role in the total fitness, compared with the reverse transcription and transcription correlation values ($R^2 = 0.26$ and 3×10^{-5}, respectively). Overall, it appears as though the relative influence of the three components of fitness is ligation>reverse transcription>forward transcription. This is interesting in that the catalytic function of the ribozymes themselves (ligation) outweighs, but does not completely dominate, the contributions by the protein enzymes in the cycle to the overall fitness of each genotype.

Evolutionary Implications

The components of fitness used here correspond to the different enzymatic steps occurring during the CE cycle; however, other components of fitness could be considered in further studies. For example, substrate affinity, rate of folding, the probability of survival, and cohort size are all potential influences. The substrate affinity can be approximated from the K_m value obtained from the Eadie-Hofstee plot, but we did not use different substrate concentrations. We suspect that our K_m value would be just an approximation of the real value, and hence inferring substrate affinity from an approximated value would be too speculative. For this particular study, we posit that the folding rate for most of these ligases (except A27) should be very similar because their mutations occurred at an unpaired region, towards the 3′ end of the ligase B16–19 (Figure 1A). Ligase A27 and the other poly-A mutations are more likely to have a different folding rate because of the excess length added, and the folding rate would be inversely related to the number of extra As added. The genotypic relatedness of the ligases could be used as an estimate of their survival probability and/or cohort size. Ligases that have lower Hamming distances to other ligases in the same generation are more likely to be recovered if they are lost by chance, and their cohort can quickly increase by point mutations from any of the closely related ligases. We measured the Hamming distances of the ligases that formed quasispecies (see Table 1 in [24]), but for the other ligases this measurement is meaningless, *e.g.*, the poly-A mutants.

We should mention that although the ligation rates could more accurately be measured with a stopped-flow device, our rate measurements were as accurate as possible by manual pipetting, and the fitness calculated are a true approximation of the dynamics of the ligases in the different enzymatic steps. Our measurements of fitness offer a more comprehensive view of the complexity of this parameter, often simplified by a single measurement. For example, if we had inferred the total fitness based on ligation rates alone, we would have concluded that 6H35 was the second-best ligase, while in reality it ranks fourth (Table 2). Interestingly, 6H35 has the lowest rate for transcription. The T7 RNA polymerase does not transcribe 6H35 as fast as it does transcribe other genotypes; it is transcribed at a speed almost half of A27, and its sequence may contain a moderate pause site for T7 RNAP.

This study demonstrates how different components of fitness can differentially affect the fate of ribozymes evolving *in vitro* (Figure 6), as it happens *in vivo* with organisms. Different fitness components can evolve to become more important under different environmental conditions. Fitness is very important to the survival of the species and its measurement should reflect its complexity. Having a colorful plumage to attract female birds, or the biggest horns to combat competitors may not be a good indicator of high fitness in every scenario. Similarly, having a high fitness on a specific component of fitness may not be beneficial for the total fitness, as the interaction of the fitness components may be positive or negative. For example, having a higher rate of reverse transcription than ligation (*e.g.*, A27, 6L22) is detrimental on the total fitness because ligases would be reverse transcribed before they have a chance to ligate the substrate to themselves, and thus will not be recognized by the RNA polymerase. The evolutionary outcome of the populations where these two mutants emerged was determined by the mutation rate. The populations were A27 emerged became extinct, whereas the populations were 6L22 did not as a consequence of the rapid emergence of 6H35 (Figures 2, 3 in [24]) with a higher fitness value (Table 2). Similar speculations about the effects of various steps of in vitro selection process on the RNA sequences that emerge after several rounds have been put forth previously for group I introns [34]. In that study, an additional possible component of total fitness was a PCR reaction, which could favor shorter sequences. In summary, to consider the components of fitness during evolution *in vitro* is not only possible but provides important insights into the genotype frequency changes that are observed in such experiments.

Acknowledgments

We thank D. Gofreed for technical assistance during this study.

Author Contributions

Conceived and designed the experiments: CDA NL. Performed the experiments: CDA. Analyzed the data: CDA NL. Wrote the paper: CDA NL.

References

1. Eigen M (1971) Selforganization of matter and the evolution of biological macromolecules. Naturwissenschaften 58: 465–523.
2. Takeuchi N, Hogeweg P (2012) Evolutionary dynamics of RNA-like replicator systems: A bioinformatic approach to the origin of life. Physics of Life Reviews 9: 219–293.
3. Joyce GF (2004) Directed evolution of nucleic acid enzymes. Annu Rev Biochem 73: 791–836.
4. Joyce GF (2007) Forty years of in vitro evolution. Angew Chem Int Ed 46: 6420–6436.
5. Díaz Arenas C, Lehman N (2009) Darwin's concepts in a test tube: Parallels between organismal and in vitro evolution. Int J Biochem Cell Biol 41: 266–273.
6. Falconer DS (1981) *Introduction to Quantitative Genetics*. London: Longman House.
7. Pearson K (1903) Mathematical contributions to the theory of evolution. XI. On the influence of natural selection on the variability and correlation of organs. Phil Trans Roy Soc Lond 200: 1–66.
8. Conner JK, Hartl DL (2004) *A Primer of Ecological Genetics*. Sunderland, MA: Sinauer.
9. Arnold SJ, Wade MJ (1984) On the measurements of natural and sexual selection: Theory. Evolution 38: 709–719.
10. Lande R (1979) Quantitative genetic analysis of multivariate evolution, applied to brain: Body size allometry. Evolution: 402–416.
11. Lande R, Arnold SJ (1983) The measurement of selection on correlated characters. Evolution 37: 1210–1226.
12. Wright MC, Joyce GF (1997) Continuous in vitro evolution of catalytic function. Science 276: 614–617.
13. Voytek SB, Joyce GF (2009) Niche partitioning in the coevolution of 2 distinct RNA enzymes Proc Natl Acad Sci USA 106: 7780–7785.
14. Joyce GF (1989) Amplification, mutation and selection of catalytic RNA. Gene 82: 83–87.
15. Lehman N, Donne MD, West M, Dewey TG (2000) The genotypic landscape during in vitro evolution of a catalytic RNA: implications for phenotypic buffering. J Mol Evol 50: 481–490.
16. Kun Á, Santos M, Szathmáry E (2005) Real ribozymes suggest a relaxed error threshold. Nature Genetics 37: 1008–1011.
17. Preston BD (1996) Error-prone retrotransposition: Rime of the ancient mutators. Proc Natl Acad Sci USA 93: 7427–7431.
18. Schmitt T, Lehman N (1999) Non-unity molecular heritability demonstrated by continuous evolution in vitro. Chem Biol 6: 857–869.
19. Ordoukhanian P, Joyce GF (1999) A molecular description of the evolution of resistance. Chem Biol 6: 881–889.
20. Lehman N (2004) Assessing the likelihood of recurrence during RNA evolution in vitro. Artif Life 10: 1–22.
21. Bisswanger H (2008) *Enzyme Kinetics: Principles and Methods*. Hoboken, NJ: Wiley-VCH.
22. Bergman NH, Johnston WK, Bartel DP (2000) Kinetic framework for ligation by an efficient RNA ligase ribozyme. Biochemistry 39: 3115–3123.
23. Díaz Arenas C, Lehman N (2010) The continuous evolution *in vitro* technique. In: Herdewijn P, editor. Current Protocols in Nucleic Acid Chemistry: John Wiley & Sons. 9.7.1–9.1.17.
24. Díaz Arenas C, Lehman N (2010) Quasispecies-like behavior observed in catalytic RNA populations evolving in a test tube. BMC Evol Biol 10: 80.
25. Burton AS, Madix RA, Vaidya N, Riley CA, Hayden EJ, et al. (2009) Gel purification of radiolabeled nucleic acids via phosphorimaging: Dip-N-Dot. Anal Biochem 388: 351–352.
26. Soll SJ, Díaz Arenas C, Lehman N (2007) Accumulation of deleterious mutations in abiotic populations of RNA. Genetics 175: 267–275.
27. Wilke CO, Wang JL, Ofria C, Lenski RE, Adami C (2001) Evolution of digital organisms at high mutational rate leads to survival of the flattest. Nature 412: 331–333.
28. Codoñer FM, Daros J–A, Sole RV, Elena SF (2006) The fittest versus the flattest: Experimental confirmation of the quasispecies effect with subviral pathogens. PLoS Pathogens 2: e136.
29. Sardanyes J, Elena SF, Sole RV (2008) Simple quasispecies models for the survival-of-the-flattest effect: The role of space. J Theor Biol 250: 560–568.
30. El-Deiry WS, Downey KM, So AG (1984) Molecular mechanisms of manganese mutagenesis. Proc Natl Acad Sci U S A 81: 7378–7382.
31. Lazcano A, Valverde V, Hernandez G, Gariglio P, Fox GE, et al. (1992) On the early emergence of reverse transcription: theoretical basis and experimental evidence. J Mol Evol 35: 524–536.
32. Ji JP, Loeb LA (1992) Fidelity of HIV-1 reverse transcriptase copying RNA in vitro. Biochemistry 31: 954–958.
33. Vartanian JP, Sala M, Henry M, Wain-Hobson S, Meyerhans A (1999) Manganese cations increase the mutation rate of human immunodeficiency virus type 1 *ex vivo*. J Gen Virol 80: 1983–1986.
34. Meluzzi D, Olson KE, Dolan GF, Gaurav A, Müller UF (2012) Computational prediction of efficient splice sites for trans-splicing ribozymes. RNA 18: 590–602.
